# Commentary on “Can Posterior Pericardial Incision Truly Improve Postoperative Complications After Cardiac Surgery?”

**DOI:** 10.21470/1678-9741-2025-0164

**Published:** 2025-12-10

**Authors:** Fatima Sohail, Shan e Ali Shoukat, Basit Ali

**Affiliations:** 1 Jinnah Sindh Medical University, Karachi, Pakistan drfatimasohailjsmuu@gmail.com

Dear Editor,

The article "Can Posterior Pericardial Incision Truly Improve Postoperative Complications After Cardiac Surgery?", published in the Brazilian Journal of Cardiovascular Surgery (or BJCVS) as "a systematic review and meta-analysis", caught our focus. Rising on days 2 - 3 is a frequent surgical condition happening within the 10 - 65% range named postoperative atrial fibrillation (POAF)^[1]^. As postoperative fluid usually accumulates in the pericardial space, standard chest tubes are less likely to infiltrate posterior effusions behind the heart, which intensifies the risk of complications that involve tamponade. Posterior pericardial effusions may progress to tamponade in the left atrium, assisting POAF^[2]^. This study, comprising a meta-analysis of 14 randomized controlled trials (RCTs) (2,275 patients), found that posterior pericardiotomy (PP) profoundly drops postoperative effusion and POAF^[3]^.

It is essential to examine the history and methodology of PP to comprehend its therapeutic relevance. Beginning in 1995, an incision in the posterior pericardium directed pericardial fluid in the left pleural cavity^[4]^. PP implies a 4-cm longitudinal incision crossing the inferior pulmonary vein to the diaphragm, parallel and posterior to the left phrenic nerve^[2]^. Several other studies have been conducted to demonstrate the efficacy of this surgical intervention. A meta-analysis of 18 RCTs (3,531 patients) showed that PP *vs.* no intervention substantially depresses POAF, tamponade, and both early and late effusions^[4]^. In a trial of 2,168 coronary artery bypass grafting patients at the Royal Hobart Hospital (2008 - 2022), PP noticeably lowers POAF and tamponade compared to controls^[5]^. In the RCT of 420 cardiac surgery patients (2017 - 2021), the posterior left pericardiotomy group showed exceptionally reduced rates of POAF and pericardial effusion compared to controls^[6]^. There remain lots of unresolved concerns regarding PP, particularly considering the long-term consequences involving heart function, arrhythmia occurrence, and patient well-being in living. To accommodate research gaps, massive, multinational RCTs firmly established strategies that are essential, as they include an expanded spectrum of the population undergoing evaluation, including young children and people who live in middleto lower-income countries.

The assurance of posterior pericardial incision as a cure for atrial fibrillation along with related disorders is emphasized in this article, specifically because of its ability to encourage fluid outflow as well as minimize pericardial effusions, each of which increases the consequences of surgery. Several questions persist, however, particularly about its prolonged impact on heart function, recurrence of challenges, and quality of life. Additional investigation is needed to investigate potential negative consequences, especially those related to higher coagulability, and involves a wider range of patient statistics, including children and people from low-resource environments, to properly understand its wider consequences.

## Artificial Intelligence Usage

The authors declare that no artificial intelligence tool was used in the preparation of this letter.

## References

[1] Abdelaziz A, Hafez AH, Elaraby A, Roshdy MR, Abdelaziz M, Eltobgy MA (2023). Posterior pericardiotomy for the prevention of atrial
fibrillation after cardiac surgery: a systematic review and meta-analysis of
25 randomised controlled trials. EuroIntervention.

[2] San TMM, Han KPP, Ismail M, Thu LM, Thet MS. (2024). Pericardiotomy and atrial fibrillation after isolated coronary
artery bypass grafting: a systematic review and meta-analysis of 16
randomised controlled trials. Cardiovasc Revasc Med.

[3] Shen ZA, Hou Y, Yu L, Wang X, Dong A, Kong M (2023). Can posterior pericardial incision truly improve postoperative
complications after cardiac surgery? a systematic review and
meta-analysis. Braz J Cardiovasc Surg.

[4] Soletti GJ, Perezgrovas-Olaria R, Harik L, Rahouma M, Dimagli A, Alzghari T (2022). Effect of posterior pericardiotomy in cardiac surgery: a
systematic review and meta-analysis of randomized controlled
trials. Front Cardiovasc Med.

[5] Rathnayake A, Goh SS, Fenton C, Hardikar A. (2024). Posterior pericardiotomy and the prevention of post-operative
atrial fibrillation and cardiac tamponade in isolated coronary artery bypass
grafting - a retrospective analysis. J Cardiothorac Surg.

[6] Gaudino M, Sanna T, Ballman KV, Robinson NB, Hameed I, Audisio K (2021). Posterior left pericardiotomy for the prevention of atrial
fibrillation after cardiac surgery: an adaptive, single-centre,
single-blind, randomised, controlled trial. Lancet.

